# Noninvasive Neurostimulation to the Inferior Frontal Sulcus or Cerebellum Improves Accuracy But Not Timing in a Speech Motor Learning Task

**DOI:** 10.1162/NOL.a.18

**Published:** 2025-09-24

**Authors:** Hannah P. Rowe, Hilary E. Miller, Rebecca M. Belisle, Tyler K. Perrachione, Frank H. Guenther

**Affiliations:** Department of Communication Sciences and Disorders, Northeastern University, Boston, MA, USA; Department of Speech, Language, and Hearing Sciences, Boston University, Boston, MA, USA; Department of Biomedical Engineering, Boston University, Boston, MA, USA

**Keywords:** non-native consonant clusters, speech motor learning, speech production, transcranial direct current stimulation

## Abstract

Speech motor learning depends on phonological working memory mechanisms in left posterior inferior frontal sulcus (LpIFS) and motor learning mechanisms in right cerebellum (RCbm). This study examines how transcranial direct current stimulation (tDCS), applied focally to these regions, affects speech production speed and accuracy when practicing novel syllables containing non-native consonant clusters. Healthy young adults (*N* = 105) practiced producing four pseudowords containing non-native clusters (e.g., “vzutp”) with either anodal stimulation to LpIFS, anodal stimulation to RCbm, or no stimulation (i.e., sham; *n* = 35 participants per condition). Trained stimuli were produced more quickly and accurately than novel stimuli across all stimulation conditions. Overall accuracy (novel and trained combined) was higher after LpIFS stimulation compared to sham but not significantly different between LpIFS and RCbm stimulation, nor after RCbm stimulation compared to sham. The difference in accuracy between novel and trained stimuli (i.e., trained − novel accuracy) was higher after either LpIFS or RCbm stimulation compared to sham, indicating better learning of syllabic motor programs with excitation of these brain regions. No significant difference in trained − novel accuracy was found between LpIFS and RCbm stimulation. Our measure of speed (i.e., stimulus duration) was not differentially affected by stimulation condition. Taken together, these findings highlight the distinct contributions of phonological and motor learning mechanisms to non-native syllable acquisition and suggest that neural stimulation can enhance learning outcomes. Future research should explore the long-term retention of these effects and examine whether real-time feedback enhances learning outcomes.

## INTRODUCTION

Speech motor learning is a crucial skill for developing and maintaining fluent speech across the life-span. Upon acquiring their native language, typical adults will almost always have fast and accurate speech planning and coordination. However, when faced with unfamiliar speech production demands, such as producing novel sound structures found in a foreign language (e.g., saying “gvazf,” where both the “gv” and “zf” consonant clusters are phonotactically illegal in English), speech becomes slower and filled with errors (Altenberg, 2005; Major, 1999). With practice, however, productions become quicker and more accurate (Buchwald et al., 2019; Buchwald & Cheng, 2023; Cheng & Buchwald, 2021; Hansen, 2001; Major, 1999; Masapollo et al., 2021; Segawa et al., 2019). The learning processes involved in these improvements likely involve both phonological and motoric components.

The gradient order directions into velocities of articulators (GODIVA) model provides a computational and neural account of the production of speech sequences, such as the sequence of phonemes in a novel syllable (Bohland et al., 2010; Guenther, 2016). According to GODIVA, upcoming phonological material is temporarily stored in a *phonological content buffer* hypothesized to reside in left posterior inferior frontal sulcus (pIFS). The model further posits that motor programs for frequently produced phoneme sequences become stored in left ventral premotor cortex (vPMC) through motor learning processes that heavily involve right hemisphere cerebellum. Projections from pIFS to left vPMC (both directly and via subcortical structures) in effect “choose” the best motor program for producing the upcoming sequence of phonemes.

According to GODIVA, a novel syllable such as “gvasf” is initially represented as five distinct phonemes in the left pIFS phonological working memory (PWM), and no optimized motor programs exist for producing the onset cluster “gv,” the offset (coda) cluster “sf,” or the full syllable “gvasf.” Due to the lack of optimized motor programs, as well as the need to properly sequence through five distinct items in PWM, productions are error-prone and relatively slow. With practice producing the novel syllable, two types of learning occur in parallel: (i) the two consonants in each cluster get “chunked” into a single onset chunk and offset chunk in PWM, thereby reducing working memory (WM) load in left pIFS, and (ii) new motor programs are learned for the consonant cluster chunks as well as for the overall sequence “gvasf,” thereby reducing the overall processing load in vPMC, as well as in RCbm, which plays a central role in learning optimized muscle activation patterns for commonly produced phoneme sequences. These reductions in processing demands lead to increases in performance accuracy. Furthermore, movement durations decrease due to (i) reduced length of the sequence stored in PWM (cf. Sternberg et al., 1978) and/or (ii) optimization of the motor program for producing the syllable. These model-based hypotheses are supported by prior functional magnetic resonance imaging (fMRI) findings, which demonstrate decreased activation in left pIFS, left vPMC, and right Cbm after practicing non-native consonant clusters (Masapollo et al., 2021; Segawa et al., 2015).

The current study used anodal transcranial direct current stimulation (tDCS) applied during the learning of novel non-native syllables to further test and refine this account. Anodal tDCS is believed to reduce the resting membrane potential, making depolarization more likely to occur under the stimulated area, potentially enhancing neural excitability and influencing motor learning processes. Although tDCS is a widely used, non-invasive brain stimulation technique, its effects across motor and cognitive domains have been shown to be inconsistent, with many studies reporting null results or variable outcomes depending on task design, stimulation parameters, and individual differences (e.g., Jalali et al., 2017; Klaus & Hartwigsen, 2020; Nettekoven et al., 2022; Westwood et al., 2017; Wiltshire & Watkins, 2020). Consequently, while tDCS holds promise as a tool for probing brain-behavior relationships, its application to speech motor learning should be interpreted with caution and understood within a broader framework that emphasizes replication and mechanistic clarity.

Several recent studies, however, suggest that tDCS can positively influence non-speech motor sequence learning (Greeley et al., 2022; Keitel et al., 2018; Shimizu et al., 2017) and other aspects of speech processing (Choi & Perrachione, 2019; Heimrath et al., 2016; Lametti et al., 2016, 2018). More relevant to the current study, prior work has examined the effects of tDCS on speech motor learning (Behroozmand et al., 2020; Buchwald et al., 2019; Deroche et al., 2017; Scott et al., 2020). Scott and colleagues (2020) found that anodal tDCS over speech motor cortex enhanced sensorimotor adaptation to altered auditory feedback, increasing the rate at which speakers adjust their productions in response to feedback perturbations, while Buchwald and colleagues (2019) demonstrated that tDCS can facilitate the production of difficult phoneme sequences, suggesting that stimulation may support the acquisition of novel speech motor patterns. Additional evidence from Deroche and colleagues (2017) and Behroozmand and colleagues (2020) indicates that brain stimulation can modulate both the accuracy and efficiency of speech motor learning, with effects observed on both behavioral performance and neural measures of adaptation.

While the aforementioned studies demonstrated improvements in speech motor learning, they did not directly dissociate the mechanistic contributions to learning of PWM and motor program optimization. Moreover, prior work has largely relied on conventional tDCS montages, which lack the spatial specificity required to selectively target cortical or subcortical substrates. The current study aims to address these limitations by employing high-definition tDCS (HD-tDCS) to more precisely stimulate two theoretically motivated brain regions: LpIFS and RCbm, during the learning of non-native syllables. This approach allows us to directly test the distinct roles of phonological chunking and motor program optimization in speech sequence learning, deepening our mechanistic understanding of how these processes contribute to improvements in both accuracy and speed during novel speech production.

Collectively, the literature to date suggests that tDCS may enhance aspects of speech motor learning, particularly in the context of sensorimotor adaptation and the production of challenging speech sounds; however, findings remain mixed, and several studies have failed to observe significant effects, underscoring the need for continued replication efforts and a clearer understanding of the conditions under which tDCS yields reliable outcomes.

In the present study, we targeted LpIFS to influence PWM-related mechanisms and RCbm to influence motor learning mechanisms. If improvements in accuracy occur only with anodal stimulation to LpIFS, this would implicate phonological processes—such as the chunking of novel consonant clusters—as the primary sources of performance improvements. Conversely, if accuracy improvements are observed with stimulation to RCbm but not to LpIFS, this would point toward a motor learning basis for those gains. Observing effects across both stimulation sites would support a combined contribution of phonological and motor learning processes. Similarly, reductions in syllable duration following LpIFS stimulation would be consistent with decreased PWM load, whereas reductions in duration following RCbm stimulation would suggest that more efficient motor program encoding underlies performance improvements.

## MATERIALS AND METHODS

### Participants

A total of 105 healthy controls (mean age: 21.45 ± 2.54, 26 male, 77 female, and 2 nonbinary individuals) were included in the study. All participants had normal hearing (verified by a hearing screening with 250, 500, 1000, 2000, 4000, 6000, and 8000 Hz tones administered bilaterally at 25 dB), reported normal or corrected-to-normal vision, were monolingual native North American English speakers, had only formal classroom experience in another language (if any), had no exposure to languages in which the non-native consonant clusters are attested, and had no reported history of psychiatric or neurological conditions known to impact speech. Participants also completed the Edinburgh Handedness Inventory. Three pre-experiment tests were administered to control for potential differences in WM (described below). The study was approved by the Boston University Institutional Review Board. Signed informed consent was obtained prior to participation. This study was not pre-registered. See Table 1 for participant demographics and mean pre-experiment testing scores.

**Table 1.** Participant demographics and mean pre-experiment testing scores

Condition	*N*	Age	Sex	Handedness	Memory for digits score (range = 0–24)	Nonword repetition score (range = 0–18)	Silent pairs score (range = 0–18)
RCbm	35	22.2 ± 2.59	27 F, 8 M	2 L, 32 R, 1 A	12.4 ± 2.83	7.9 ± 2.39	13.8 ± 4.48
LpIFS	35	20.9 ± 2.53	27 F, 8 M	0 L, 31 R, 4 A	12.5 ± 3.85	8.23 ± 3.14	14.7 ± 4.38
Sham	35	21.2 ± 2.39	23 F, 10 M, 2 NB	3 L, 31 R, 1 A	12.8 ± 3.19	8.2 ± 3.05	14.5 ± 4.06

*Note*. RCbm = right cerebellum, LpIFS = left posterior interior frontal sulcus, F = female, M = male, NB = nonbinary, L = left, R = right, A = ambidextrous.

### Pre-Experiment Testing

For all pre-experiment testing, participants were seated in front of a desktop computer in a soundproof booth. Stimuli were auditorily presented at a comfortable loudness level. Following the offset of each stimulus presentation, participants saw a blank screen with a white cross. The cross then turned green, at which point the participant was presented with a tone for 50 ms that acted as a GO cue indicating that the participant should speak the stimulus. Productions were recorded at 44.1 kHz using a Shure MX153 earset microphone, Behringer Ultragain Pro two-channel microphone amplifier, and Roland Quad Capture sound card.

To obtain experiment-independent measures of WM capacity, participants completed three WM-related tasks. The first task was the Memory for Digits subtest from the Comprehensive Test of Phonological Processing (CTOPP; Bruno & Walker, 1999), in which participants heard recordings of a string of numbers of increasing length (three to ten digits) and were asked to repeat the digits in the order they heard them. Because the phonological material (i.e., the digit names) is highly overlearned, this task primarily probes general WM (i.e., the ability to temporarily store and manipulate various types of information, such as visual or verbal data, for complex cognitive tasks) rather than *phonological* WM (i.e., the ability to temporarily store and manipulate sound-based information, such as phonemes or syllables, for language processing and speech production) specifically. To gauge PWM, participants completed a novel nonword repetition task, in which they heard recordings of pseudowords of increasing length (three syllables to eight syllables) and were asked to repeat the stimulus exactly as they heard it and as clearly as they could after the beep. The test included three trials of each syllable length. For the digit span and nonword repetition tasks, scoring was conducted in real-time by the experimenter, who gave the participant a score of 1 if they were correct or 0 if they were incorrect on each trial. Testing was discontinued if participants scored 0s for three consecutive trials.

Finally, to assess PWM in the absence of a speech motor component, participants completed an assessment developed in-house called the Silent Pairs Task. For this task, participants first heard recordings of pseudowords of increasing length (three syllables to eight syllables) followed by recordings of three pairs of syllables. All of the pairs of syllables were in the stimulus, but two of them were not in the correct order (e.g., the participant hears: “see lo ra,” followed by the three options: “lo see,” “see ra,” “lo ra”). Participants were instructed to press either “1,” “2,” or “3” followed by “enter” on the keyboard to indicate which syllable pair was in the stimulus *and* in the correct contiguous order. Scoring was conducted in real-time automatically using a MATLAB script, which gave the participant a score of 1 if they were correct or 0 if they were incorrect. Because participants had a 33% chance of guessing correctly on each item (making it unlikely to get consecutive incorrect answers needed to establish a cutoff), all participants completed the full assessment.

### Stimuli

For the speech motor learning task, speech stimuli consisted of two sets (Set A and Set B) of 14 CCVCC pseudowords (Segawa et al., 2015), which were counterbalanced across participants such that approximately half of the participants within each condition learned Set A and the other half within each condition learned Set B. For each set, there were four training stimuli and 10 testing stimuli (Table 2), all of which contained a non-native syllable-initial (onset) and syllable-final (coda) consonant cluster. Non-native consonant clusters are clusters that occur in some natural human languages (e.g., Hebrew, Russian, Romani, Polish, Lithuanian) but are phonotactically illegal in English. Stimuli were selected based on prior studies (Masapollo et al., 2021, 2023; Segawa et al., 2015, 2019) to probe articulatory learning of novel sequences beyond familiar phonotactic patterns. While some of the clusters selected may not be naturally occurring in human languages (e.g., /vb/, /tp/), all stimuli were produced by a trained human speaker and perceptually validated to ensure they were intelligible and producible, reflecting the range of articulatory capabilities within the human speech motor system. None of the stimuli were orthographic or phonological words according to the MRC Psycholinguistic Database (Coltheart, 1981). Each consonant cluster appeared in only one stimulus; that is, each stimulus had a unique onset cluster and coda cluster.

**Table 2.** Training and testing stimuli for Set A and Set B

Stimulus set	Training	Testing	
Set A	fpashp	dzukf	shkizg
	fshapf	kshapk	tfeshch
	vbimk	kpimch	fsefk
	zvusch	vzutp	bdechk
		zdebg	vgamsh

Set B	dzukf	fpashp	tfeshch
	kpimch	vbimk	fsefk
	kshapk	fshapf	bdechk
	vzutp	zvusch	vgamsh
		shkizg	zdebg

The auditory models used in this study were utilized in prior published work (Masapollo et al., 2021, 2023; Segawa et al., 2015, 2019). Stimuli were recorded by a phonetically trained female native speaker of American English in a sound-attenuated booth. A phonetically trained female native speaker of American English was recorded producing each nonword. The speaker practiced the target sequences until they could be produced fluently without errors such as epenthesis, consonant deletion, or substitutions. Multiple randomized repetitions of each token were recorded, and one instance per token was selected based on clarity and acoustic similarity to other stimuli in the set. These auditory models were then used in the training and test phases, with perceptual validation ensuring that the stimuli were error-free and accurately represented the intended phonotactic sequences. Productions were recorded using Audacity (Version 2.0.3; Audacity Team) via a professional-quality microphone (Samson C01U studio condenser) connected to a preamplifier (44.1-kHz sampling rate, 32-bit quantization). All productions were matched for peak intensity and duration (i.e., 480 ms) using Praat (Boersma, 2014).

### Speech Motor Learning Procedure

The experimental protocol involved the completion of a cued syllable production task (Segawa et al., 2015, 2019). In this version of cued syllable production, participants repeated pseudowords that contained non-native consonant clusters. For all three phases outlined below (familiarization, training, and testing), participants were seated in front of the same computer as during the pre-experiment testing in the soundproof booth. Participants were instructed, “On each trial, silently read and listen to the stimulus. When you hear the beep, say the target stimulus as quickly and accurately as possible.” Stimuli were presented over loudspeakers at a comfortable loudness level and visually on the computer monitor using orthographic transcriptions (see Masapollo et al., 2021, for rationale on the use of both auditory and visual modalities). Similar to the pre-experiment testing, following the offset of each stimulus presentation, participants saw a blank screen with a white cross. The cross then turned green (after a randomly jittered delay between 500 and 1,500 ms), at which point the participant was presented with a tone for 50 ms that acted as a GO cue for them to repeat the syllable aloud. Productions were again recorded at 44.1 kHz using the same audio setup as described in the Pre-Experiment Testing section above.

#### Familiarization (∼2 min)

To familiarize participants with the task, they were presented with monosyllabic pseudowords with native and non-native consonant clusters (i.e., “flisk,” “prunge,” “dgamj,” and “gvuchb”). Using the feedback procedure of Buchwald and colleagues (2019), the participant was provided with feedback to improve their knowledge of results (i.e., whether the target was produced accurately) and their knowledge of performance (i.e., what aspects of the production were in error, when applicable). The feedback was provided only when the participant produced the target inaccurately.

#### Training (∼40 min)

For the training phase, participants produced four syllables (72 repetitions each) with distinct non-native consonant clusters. Training was divided into two runs of six blocks (24 trials of stimulus repetitions per block), with a 1-minute break in between runs. Stimulus presentation order was pseudorandomized with the constraint that no stimulus was presented more than twice in a row. Participants did not receive feedback during this phase. Application of stimulation (or sham stimulation) occurred during the first 20 minutes of the training phase, as described below.

#### Testing (∼20 min)

For the testing phase, participants were tested on their ability to produce the learned stimuli from the training phase, as well as their ability to produce novel stimuli with untrained non-native consonant clusters. Participants produced 80 trials of learned stimuli (20 repetitions each of the 4 learned stimuli from the training phase) and 80 trials of novel stimuli with untrained non-native clusters (8 repetitions each of 10 novel stimuli). Testing was divided into two four-block runs (20 trials of stimulus repetitions per block) with a 1-minute break in between. Similar to the training phase, stimulus presentation order was randomized, and participants did not receive feedback.

In this study, learning was operationalized as improved performance on trained (practiced) stimuli relative to untrained (unseen) stimuli during the post-training test phase. This cross-sectional comparison allowed us to assess whether exposure to specific items during training led to more accurate or fluent production, and whether this effect was amplified under stimulation versus sham. Therefore, untrained items served as a control condition to help isolate item-specific learning effects. To reduce the likelihood of generalization, untrained stimuli were designed to differ in segmental composition (e.g., stop–stop or stop–fricative clusters) from trained items (e.g., fricative–stop or fricative–fricative clusters). We calculated difference scores (trained − untrained) for both accuracy and duration (measures defined below) as a summary index of learning, controlling for non-specific performance factors such as task exposure or fatigue. This approach aligns with prior studies using similar paradigms (Masapollo et al., 2021, 2023; Segawa et al., 2015, 2019) and helps dissociate learning from general task familiarity. Finally, to mitigate potential item-level confounds, stimuli were counterbalanced across participants such that each item appeared in both trained and untrained conditions across the sample.

### tDCS Procedure

**Figure 1.**
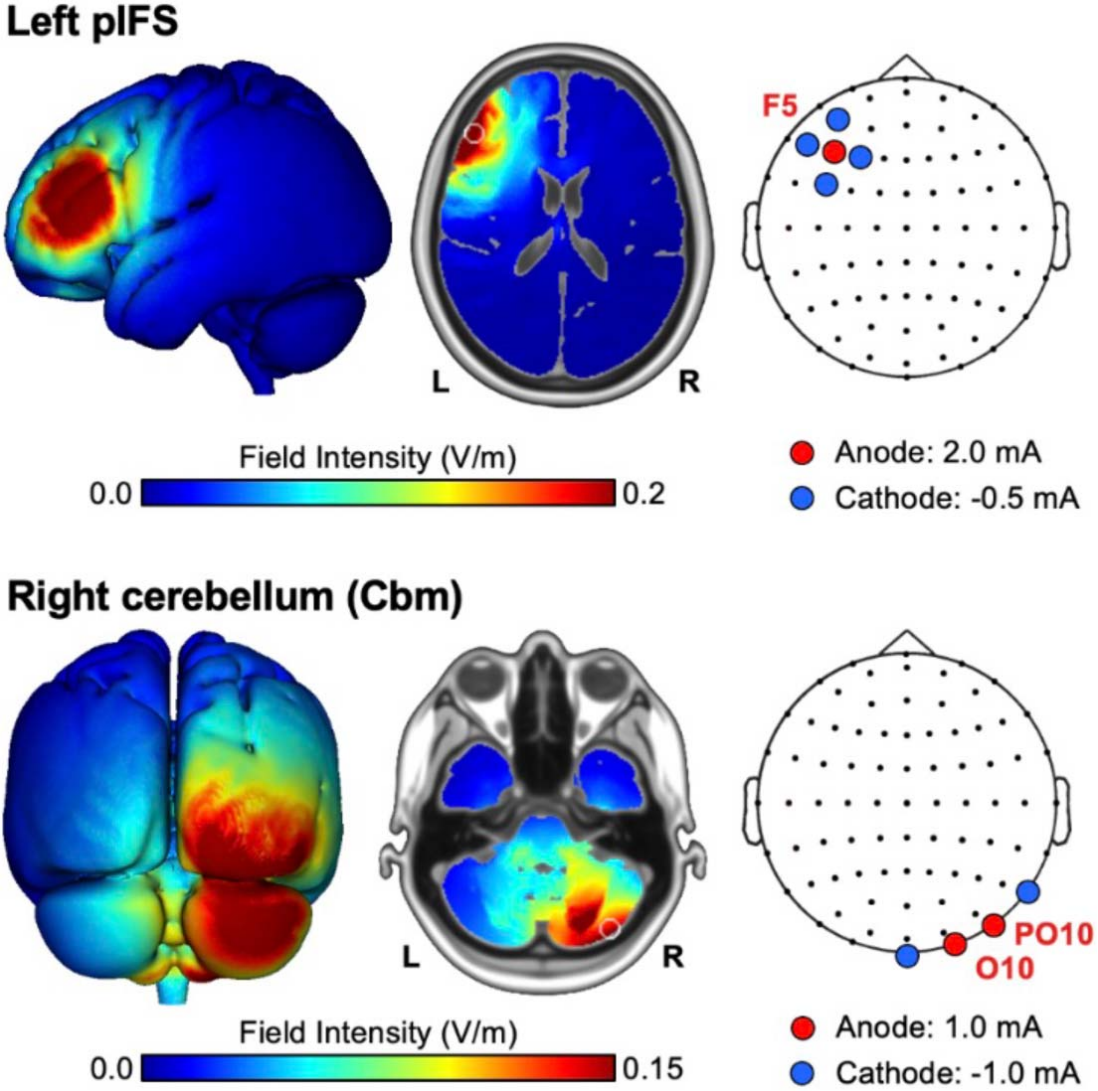
Electrode montages and simulated electrical field intensities for the LpIFS (top) and RCbm (bottom) stimulation conditions. LpIFS = left posterior interior frontal sulcus, RCbm = right cerebellum.

Neurostimulation was delivered using a Soterix M×N HD-tDCS system. In contrast to traditional tDCS systems, HD-tDCS offers more focal stimulation to target brain regions, which improves replicability across studies (Masina et al., 2021). Participants were randomly assigned to one of three conditions: (1) anodal RCbm, (2) anodal LpIFS, or (3) sham. In both the stimulation and sham conditions, the experimenter ensured that the resistance of each channel was less than 20 kΩ (Khadka et al., 2020). Electrode montages (using the 10–10 system) were selected based on simulations using the HD-Explore software (Soterix Medical Inc.; Datta et al., 2009; Huang et al., 2017) to optimize field intensity and current flow to the regions of interest (Figure 1). Anodes (positively charged stimulating electrodes) for the RCbm condition were placed at O10 and PO10, and cathodes (negatively charged electrodes) were placed at P10 and Iz (Datta et al., 2009; Kuo et al., 2013). The anode for the LpIFS condition was placed at F5, while the cathodes were placed at F7, FC5, AF7, and F3.

Prior studies have differed regarding the timing of stimulation relative to performance of the behavioral task, with some stimulating for 20 minutes immediately prior to the behavioral task and others stimulating during the behavioral task (see Buchwald et al., 2019). To maximize the likelihood of detecting an effect of stimulation on behavioral performance given the 20-minute limit for stimulation, participants performed the behavioral task both during stimulation and for 20 minutes after stimulation ceased. For the RCbm and LpIFS conditions, there was a 30-second linear ramp from 0 to 2 mA, with tonic 2 mA stimulation for the first 20 minutes of the training phase.

For the sham condition, the electrode montage, which was identical to either the RCbm or LpIFS montage, was randomly assigned across subjects and counterbalanced to ensure approximate equal representation of each montage. The sham condition involved the same ramp-up from 0 to 2 mA, but this was immediately followed by a ramp-down to 0 mA just before the behavioral task began. The study was single-blind, with participants unaware of their assigned stimulation condition. Prior work using this procedure (Ambrus et al., 2012) has found that participants are unable to detect whether they were receiving anodal or sham stimulation and, therefore, were effectively blinded to condition.

### Behavioral Measures

To examine motor learning, we examined two primary measures of interest: production accuracy and duration. These metrics are commonly used to characterize learning in studies examining motor sequence learning and second language acquisition (e.g., Buchwald et al., 2019). Average values (across trials and participants) for these measures were derived during the testing phase, where we compared the trained non-native syllables to the novel non-native syllables. Given that prior studies did not find differences in reaction time between novel and trained stimuli in typical speakers (Masapollo et al., 2021; Segawa et al., 2015), we did not include reaction time in our analyses.

#### Production Accuracy

For production accuracy, a trained research assistant, who was blind to condition (LpIFS, RCbm, and sham) and stimulus familiarity (trained or novel), scored each production for the presence of the following: disfluency (i.e., repetitions, self-corrections); unrecognizable from target (i.e., due to excessive distortion or misarticulation); phoneme deletion/omission; phoneme insertion; consonant substitution; incorrect phoneme order; vocoid epenthesis (based on a visible second or higher formant in the spectrogram and a drop in intensity prior to the second consonant; Wilson et al., 2014); incorrect voicing (i.e., voiced or voiceless distinction of a consonant did not match the intended target); or vowel substitution. Scoring decisions were made based on perceptual judgment and visual cues in the spectrogram and waveform. Accuracy for each production was then scored as 1 (i.e., accurate) if they did not contain any of these errors or 0 (i.e., inaccurate) if an error was present. Productions from all participants were scored by the same rater. To obtain intra-rater reliability, the research assistant rerated the accuracy of a random selection of 15% of the participants, which represented the full set of stimuli. Intraclass correlation ICC(2, 1) demonstrated excellent intra-rater reliability (0.98 [0.98, 0.99]) for accuracy ratings.

#### Production Duration

Only productions that either had no errors or solely a vocoid epenthesis were included in the production duration analysis. Productions with epentheses were included so that improvements in duration due to the shortening of the epentheses would be evident (Davidson, 2005). For the duration analysis, a trained research assistant, who was blind to condition and stimulus familiarity, viewed the waveform and spectrogram for each stimulus and identified the threshold that the intensity must exceed or fall under to be considered the beginning or end of speech, respectively, for each participant. Therefore, a fixed intensity threshold was determined for each participant to normalize for individual speaking volume. Custom MATLAB software was then used to automatically detect the onset and offset of each stimulus. This intensity-based algorithm was used in prior studies employing the same stimuli and paradigm (Masapollo et al., 2021, 2023; Segawa et al., 2015, 2019). Measuring whole-word duration captures learning-related gains across both clusters and vowel transitions. Stimulus onset was measured from the detectable acoustic energy (typically the release of the initial stop or fricative) at the beginning of the stimulus to the offset of energy at the end of the stimulus. All trials underwent post hoc verification by a trained research assistant, who inspected the spectrograms of each trial and manually corrected misalignments when needed. If over 10% of a participant's trials required adjustment, their data were reprocessed using a recalibrated threshold to ensure consistency. Because duration is primarily scored automatically, inter-rater reliability data were not calculated.

We had four outcome measures for this study: accuracy averaged across trained and novel words (herein, *accuracy*), duration averaged across trained and novel words (herein, *duration*), the difference in accuracy between trained and novel words (herein, *accuracy difference*), and the difference in duration between trained and novel words (herein, *duration difference*). We chose to use these four metrics because the averaged measures (accuracy and duration) capture overall speech production ability, which allows us to assess whether stimulation broadly improves performance irrespective of word familiarity. In contrast, the difference measures serve as learning-specific indices (as indicated above), reflecting the extent to which participants performed better on trained versus novel items. These difference scores are therefore particularly informative for evaluating whether stimulation influenced learning and retention of specific speech forms, beyond general task performance.

Because two of our outcome measures were based on the difference in performance between trained and novel stimuli on accuracy and duration in the test phase, we sought to ensure that any observed differences were not simply due to unequal exposure during testing (i.e., 20 repetitions each of trained stimuli and 8 repetitions each of novel stimuli). Thus, we conducted a mixed-effects model with fixed effect of test-half (first vs. second) and random intercepts for participant. This analysis revealed no significant effect of test-half on duration (*β* = 0.001, *SE* = 0.003, *t* = 0.24, *p* = 0.81) nor on accuracy (*β* = 0.001, *SE* = 0.01, *t* = 0.08, *p* = 0.94), suggesting an absence of practice effects.

### Statistical Analyses

All statistical analyses were performed in R (R Core Team, 2014) using models implemented in the packages lme4 and lmerTest, and post hoc tests via emmeans. We analyzed the effects of the experimental conditions of syllable-production duration using a linear mixed effects model (LMEM). Fixed factors in the model included Condition (LpIFS, RCbm, or Sham), Familiarity (trained or novel), and their interaction. Random factors included by-participant random intercepts and random slopes for the within-subjects factor Familiarity, as well as random intercepts for Stimulus. Categorical model contrasts were sum (deviation) coded. We analyzed effects of the experimental manipulations on syllable-production accuracy a generalized linear mixed-effects model (GLMEM) for binomial data using the same model structure. Post hoc analyses for pairwise differences were conducted using estimated marginal means. For all post hoc analyses, we employed Bonferroni correction to account for multiple comparisons. However, we also report uncorrected *p* values because our analyses were guided by a hypothesis that each of the three stimulation conditions would produce a distinct pattern of effects. To control for baseline differences in pre-experiment test scores, we conducted independent samples *t* tests to compare scores across stimulation conditions. Lastly, to evaluate the relationships between pre-experiment test scores and outcome variables, we conducted Bonferroni-corrected pairwise correlations using Pearson's correlation coefficients.

To ensure sufficient statistical power for detecting meaningful effects in our study, we conducted a power analysis based on prior research. In a previous tDCS study (Scott et al., 2020) with 18 participants, anodal tDCS to the left ventral premotor cortex significantly increased learning rates in a sensorimotor adaptation task. This effect was used as the reference for our power calculation. Our analysis suggests that 35 participants per stimulation condition will provide 80% power (*α* = 0.05) to detect a change in learning rate that is 94% of the magnitude of the original effect.

## RESULTS

No participants reported adverse effects during or after the stimulation session. Although 49/105 participants (46.7%) correctly identified the stimulation condition they received, the distribution of guesses suggests a strong response bias. In the active stimulation group, 43/70 participants (61.4%) guessed correctly, while in the sham group, only 6/35 participants (17.1%) guessed correctly (*χ*^2^ = 4.03, *df* = 1, *p* = 0.05). Notably, the majority of participants in both groups—including 83% of those who received sham—guessed they had received active stimulation. This pattern indicates a general bias toward assuming active stimulation, rather than accurate detection of the stimulation condition, and complicates interpretation of blinding efficacy.

Table 3 provides the descriptive statistics (means and standard deviations) for duration and accuracy by stimulation condition and stimulus familiarity. Boxplots showing the distributions of production durations (averaged across trials for each subject) for novel and trained stimuli in each stimulation condition are included in the Supplementary Materials, available at https://doi.org/10.1162/NOL.a.18. The percentage of each error type by onset versus offset consonant cluster and condition can also be found in the Supplementary Materials.

**Table 3.** Means and standard deviations for duration (in seconds) and accuracy by stimulation condition and stimulus familiarity

		Duration		Accuracy	
		*M*	*SD*	*M*	*SD*
LpIFS	Trained	0.64	0.11	0.42	0.20
	Novel	0.65	0.09	0.37	0.17
RCbm	Trained	0.65	0.10	0.35	0.20
	Novel	0.67	0.11	0.30	0.15
Sham	Trained	0.62	0.09	0.27	0.22
	Novel	0.64	0.10	0.29	0.17

**Figure 2.**
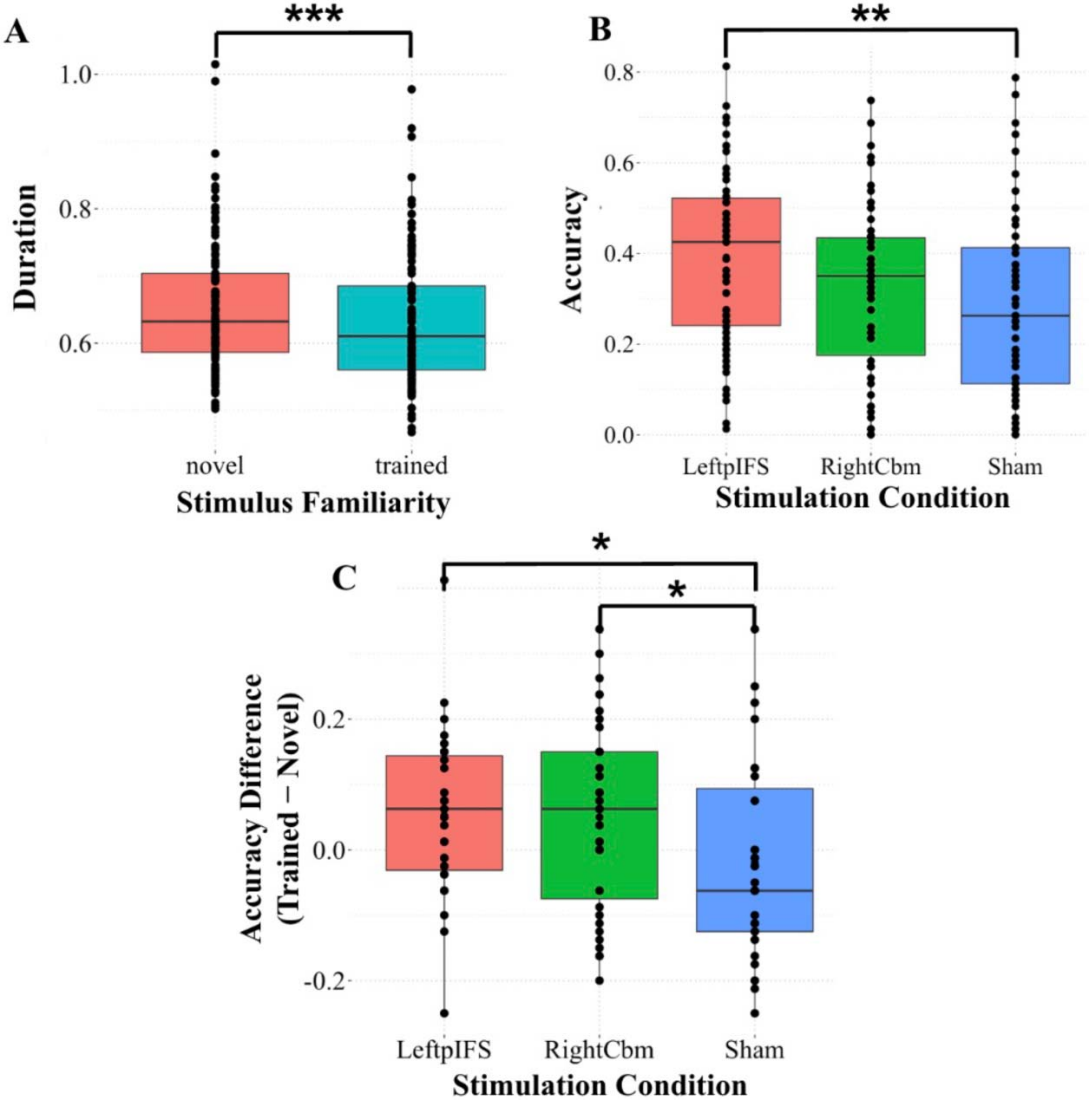
Results of post hoc tests of significant main effects and interactions from the duration and accuracy statistical models. (A) Duration as a function of stimulus familiarity (all stimulation conditions combined). (B) Accuracy as a function of stimulation condition (novel and trained stimuli combined). (C) Accuracy difference (i.e., trained stimulus accuracy − novel stimulus accuracy) as a function of stimulation condition. Asterisks indicate statistically significant pairwise comparisons (*p < 0.05, **p < 0.01, ***p < 0.001; uncorrected). Corresponding Bonferroni-corrected *p* values are reported in the text. Boxplots show the median (solid horizontal line), interquartile range (box), and whiskers extending to 1.5× the interquartile range. Individual points represent participant-level data.

The LMEM for duration revealed a significant main effect of familiarity (*F*(1) = 38.82, *p* < 0.001, *η*^2^ = 0.20). However, there was no significant main effect of condition (*F*(2) = 1.32, *p* = 0.27, *η*^2^ = 0.03) nor a significant interaction between condition and familiarity (*F*(2) = 1.56, *p* = 0.21, *η*^2^ = 0.03). A post hoc comparison for the main effect of familiarity revealed that duration was significantly shorter for trained stimuli compared to novel stimuli (*estimate* = 0.03, *SE* = 0.004, *z* = 6.23, *p* < 0.001, *d* = 0.61; panel A of Figure 2). This effect survived Bonferroni correction (adjusted alpha = 0.02).

The GLMEM for accuracy revealed a significant main effect of condition (*F*(2) = 2.36, *p* = 0.02, *η*_*p*_^2^ = 0.42) and a significant interaction between condition and familiarity (*F*(1) = 3.29, *p* = 0.03, *η*_*p*_^2^ = 0.50). Although the effect of familiarity on accuracy was not statistically significant, it was associated with a large effect size (*F*(2) = 3.15, *p* = 0.08, *η*_*p*_^2^ = 0.32). A post hoc comparison for the main effect of condition revealed that accuracy was significantly higher in the LpIFS condition compared to the sham condition (*estimate* = −0.90, *SE* = 0.34, *z* = −2.67, *p* < 0.01, *d* = −0.26; panel B of Figure 2). This effect survived Bonferroni correction (adjusted alpha = 0.02). There was no significant difference in accuracy between the LpIFS and RCbm conditions (*estimate* = 0.61, *SE* = 0.34, *z* = 1.80, *p* = 0.07, *d* = 0.18) nor between the RCbm and sham conditions (*estimate* = −0.29, *SE* = 0.34, *z* = −0.87, *p* = 0.39, *d* = −0.09). A post hoc comparison for the interaction between condition and familiarity revealed that the trained − novel difference in accuracy was significantly smaller in the sham condition compared to the LpIFS condition (*estimate* = 0.62, *SE* = 0.25, *z* = 2.45, *p* = 0.01, *d* = 0.24; panel C of Figure 2) and compared to the RCbm condition (*estimate* = 0.49, *SE* = 0.26, *z* = 1.91, *p* = 0.05, *d* = 0.19). However, only the sham to LpIFS comparison survived Bonferroni correction (adjusted alpha = 0.02). There was no significant difference in accuracy between the LpIFS and RCbm conditions (*estimate* = −0.13, *SE* = 0.25, *z* = −0.53, *p* = 0.60, *d* = −0.05).

Independent samples *t* tests revealed no significant differences in pre-experiment test scores across stimulation conditions (all *p* > 0.05). Pairwise Pearson’s correlation coefficients were calculated to investigate the relationship between pre-experiment working-memory-related test scores (Memory for Digits Task; Nonword Repetition Task; Silent Pairs Task) and duration and accuracy (Table 4). To ensure that our correlation results were not driven by extreme values, we first screened for outliers using a z-score method (threshold = |z| > 3). Any data points exceeding this threshold were excluded from the correlation analyses. After applying a Bonferroni correction for multiple comparisons (adjusted alpha = 0.016), all correlations between accuracy and the pre-experiment test scores were significant, whereas no correlations between duration and pre-experiment test scores reached significance. We also analyzed correlations with learning-specific measures (duration difference and accuracy difference; Table 5). No significant correlations were found between the pre-experiment test scores and either learning-specific measure.

**Table 4.** Pearson’s correlation coefficients investigating the relationships between pre-experiment test scores and duration and accuracy (averaged across novel and trained)

	Duration		Accuracy	
	*r*	*p*	*r*	*p*
Memory for Digits Task	0.10	0.34	0.30	0.002*
Nonword Repetition Task	0.03	0.78	0.27	0.008*
Silent Pairs Task	−0.15	0.16	0.38	>0.001*

*Note*. Significant *p* values (with Bonferroni correction) are denoted with asterisks.

**Table 5.** Pearson’s correlation coefficients investigating the relationships between pre-experiment test scores and improvements in duration and accuracy

	Duration difference (Trained − Novel)		Accuracy difference (Trained − Novel)	
	*r*	*p*	*r*	*p*
Memory for Digits Task	0.21	0.04	0.10	0.34
Nonword Repetition Task	0.09	0.37	0.09	0.35
Silent Pairs Task	−0.003	0.96	0.12	0.22

## DISCUSSION

The present study investigated the effects of anodal tDCS to LpIFS or RCbm on the production of syllables containing non-native consonant clusters in neurotypical adults. When collapsing across all stimulation conditions (i.e., LpIFS, RCbm, and sham), participants demonstrated a significant difference in duration between novel and trained stimuli, indicating robust learning effects as a whole. LpIFS stimulation yielded higher overall accuracy (across both novel and trained stimuli combined) and a larger accuracy difference compared to sham. RCbm stimulation also resulted in a larger accuracy difference compared to sham; however, this effect did not remain statistically significant after Bonferroni correction. Finally, participants with higher pre-experiment WM-related test scores exhibited better overall production accuracy. These findings are discussed in detail below.

### Increased Overall Accuracy With Stimulation to LpIFS Compared to Sham

The model for production accuracy revealed a large effect size for stimulus familiarity, suggesting that repeated production of stimuli may lead to improvements in accuracy. This trend aligns with prior behavioral studies involving phonotactically illegal syllables (Buchwald et al., 2019; Hansen, 2001; Major, 1999; Masapollo et al., 2021, 2023; Segawa et al., 2019). However, this effect did not meet statistical significance and, therefore, warrants further investigation.

Of more interest is the finding of a main effect of condition on performance accuracy. Post hoc tests revealed significantly higher production accuracies (both novel and trained stimuli combined) for LpIFS stimulation compared to sham, but not for RCbm stimulation compared to sham. According to the GODIVA model, LpIFS is hypothesized to house a phonological content buffer involved in temporarily storing upcoming phonological information. While speculative, our findings raise the possibility that stimulation to LpIFS may have subtly enhanced PWM, potentially supporting more accurate speech sequencing. However, given the lack of statistical significance and the absence of direct pre- and post-stimulation measures of PWM, this interpretation should be treated cautiously and warrants further investigation in future studies.

Unlike LpIFS stimulation, there was no significant improvement in overall accuracy for RCbm stimulation compared to sham. In the GODIVA framework, RCbm is primarily involved in learning motor programs for producing frequent phoneme sequences and does not play a role in PWM (cf. Scott & Perrachione, 2019). The lack of an increase in overall accuracy with RCbm stimulation suggests that production accuracy may depend more heavily on higher level PWM and/or sequencing mechanisms located in/near left pIFS than lower level motor learning mechanisms.

### Improved Learning With Stimulation to LpIFS and RCbm Compared to Sham

The model for production accuracy also revealed a significant interaction between stimulation condition and stimulus familiarity. Post hoc tests revealed significantly larger accuracy increases (i.e., from novel to trained stimuli) for LpIFS stimulation compared to sham as well as for RCbm stimulation compared to sham; however, the RCbm comparison did not survive Bonferroni correction, indicating that this finding should be interpreted with caution. Both LpIFS and RCbm are thought to be involved in the learning of speech sequences, such as the sequences of phonemes making up the phonotactically illegal syllables used here (Bohland et al., 2010; Bohland & Guenther, 2006; Guenther, 2016). Within the GODIVA framework (Bohland et al., 2010; Guenther, 2016), LpIFS is proposed to function as a PWM buffer that temporarily stores upcoming speech content. According to the DIVA model (Tourville & Guenther, 2011), which is another computational framework that explains how the brain coordinates speech through sensory feedback and motor planning, RCbm is implicated in optimizing motor programs for frequently produced speech sequences. Based on these models, we hypothesized that anodal stimulation to LpIFS would enhance PWM processes and thus improve overall production accuracy, while anodal stimulation to RCbm would enhance the learning of novel motor sequences, reflected in greater differences between novel and trained stimuli. Our findings partially supported these hypotheses: LpIFS stimulation improved overall production accuracy, while both LpIFS and RCbm stimulation enhanced learning. However, the absence of a significant main effect of RCbm stimulation on overall accuracy fails to provide support for its role in accuracy. Rather, it suggests that overall production accuracy may depend more heavily on higher level PWM mechanisms localized to LpIFS, as predicted by the model, but that motor learning processes involving RCbm still contribute to the acquisition of novel speech sequences. We emphasize, however, that the RCbm finding is tentative, as it did not survive Bonferroni correction, and that future studies designed to separately probe motor programming and phonological memory processes—for example, by manipulating the complexity of motor demands versus memory load—will be necessary to directly test these mechanistic hypotheses.

Notably, the sham condition showed a negative difference score for accuracy, suggesting no observable learning of the trained clusters under non-stimulated conditions. This pattern raises the possibility that the training paradigm alone may not have been sufficient to elicit measurable learning and that stimulation may have played a facilitative role in enabling, rather than merely enhancing, learning. In this context, the difference scores observed in the stimulation groups may reflect the presence of learning driven by tDCS, rather than an amplification of an existing training effect.

### No Effect of Stimulation on Movement Duration

The model for duration yielded a main effect of stimulus familiarity (with post hoc tests revealing significantly lower utterance durations for trained compared to novel stimuli), indicating that participants learned to produced stimuli more quickly with practice. This result is consistent with prior behavioral studies of phonotactically illegal syllable learning (Masapollo et al., 2021; Segawa et al., 2019). However, no main effect of stimulation condition was found, nor was there a significant interaction between condition and familiarity. This finding was counter to our a priori expectations, since we expected improvements in accuracy with stimulation to be accompanied by decreases in duration because of either (i) more efficient PWM processing with stimulation to LpIFS, or (ii) improved movement efficiency due to better motor program learning with stimulation to RCbm. Although we found that movement duration was reduced for trained compared to novel words, there was no effect of condition on this difference (i.e., no interaction between condition and familiarity). One plausible interpretation of this finding is that learning of motor programs for novel phoneme sequences occurs in two stages: (i) relatively fast improvement in producing the proper phonemes (as indicated by our accuracy findings), and (ii) a much slower process of optimizing the motor programs for these sequences over hundreds or thousands of productions (as occurs with native language syllables). Our experimental paradigm involved only 72 productions of each trained stimulus over a 40-minute period; thus, there was no opportunity for overnight consolidation. While this amount of training was long enough to reveal improvements in the relatively fast learning of non-native syllables due to stimulation, it may not have been enough to detect slower improvements in motor program efficiency. Achieving native-like articulatory coordination likely requires significantly more productions than were feasible within our study design.

### Correlations With Pretest Measures of Working Memory Capacity

To gauge the role of WM capabilities in the learning and performance of non-native syllables, participants performed three pre-experiment WM-related tests: (i) a nonword repetition task that gauges PWM, (ii) a forward digit span task meant to gauge more general WM mechanisms (albeit with a phonological component since the remembered numbers must be spoken), and (iii) a novel PWM task dubbed the Silent Pairs Task, in which participants identified which of a candidate set of syllable pairs was present in an auditorily presented multisyllabic stimulus by pressing a key. This third task was meant to probe PWM without requiring the generation of speech. As expected, we found significant correlations between overall production accuracy and all three memory scores, whereas overall movement duration did not correlate with any of the pre-experiment WM measures. Collectively, these findings indicate that better general WM capacity is associated with higher production accuracy but not with shorter utterance durations.

### Comparison to Prior Studies of the Effects of Brain Stimulation on Motor Learning

While transcranial stimulation has been explored as a tool for modulating brain activity to enhance speech motor learning, its effects across motor domains remain inconsistent and, at times, unreliable (e.g., Jalali et al., 2017; Klaus & Hartwigsen, 2020; Nettekoven et al., 2022; Westwood et al., 2017; Wiltshire & Watkins, 2020). Our findings should therefore be interpreted within this broader context—namely, that tDCS may facilitate learning under certain conditions, but more work is needed to clarify when, how, and for whom these effects occur. Within this evolving framework, our results provide additional support for the possibility that stimulation to specific brain regions—such as LpIFS—can enhance aspects of speech motor learning. Specifically, we observed improved learning (as indexed by accuracy differences between novel and trained stimuli) following stimulation to LpIFS. These findings align with prior studies targeting nearby regions (Fiori et al., 2014; Restle et al., 2012). Restle and colleagues (2012) reported increased accuracy of Japanese sentence production in German speakers following intermittent theta burst stimulation to posterior inferior frontal gyrus; the authors concluded that stimulation facilitated translation of phonological input to motor output. Similarly, Buchwald and colleagues (2019) demonstrated improvements in accuracy on non-native consonant clusters following anodal tDCS to left motor cortex. Together, these findings suggest that stimulation to frontal regions involved in phonological-motor integration may, under certain conditions, support speech motor learning—though further replication and mechanistic clarification are essential.

Our findings are also in alignment with two studies that tested the effects of anodal tDCS on sensorimotor adaptation and tongue twister production accuracy. Scott and colleagues (2020) found that anodal tDCS to left vPMC increased the rate of sensorimotor adaptation in response to altered auditory feedback; the authors suggested that tDCS to left vPMC enhanced learning of new feedforward speech motor plans. In a study on tongue twister production, Fiori and colleagues (2014) found increased accuracy with anodal tDCS to left inferior frontal sulcus.

In contrast to Fiori and colleagues (2014), Wiltshire and Watkins (2020) reported no changes in tongue twister accuracy following tDCS to left inferior frontal sulcus. In their study, participants showed an improvement in performance, but these changes did not differ between the anodal stimulation condition and sham. There are a number of possible reasons for this discrepancy in findings, one of which is the differences in study protocol; Wiltshire and Watkins (2020) used shorter tongue twisters and introduced a control task of repeating simple sentences. It is possible that the use of simpler sentences and the reduced duration of the tongue twisters diminished the effectiveness of tDCS in modulating performance.

Our findings of a larger accuracy difference under cerebellar stimulation compared to sham also align with a substantial body of literature showing that tDCS to the cerebellum enhances the accuracy of general motor skill learning (see Kumari et al., 2019, for a review); however, this result did not survive Bonferroni correction and should therefore be interpreted with caution. The cerebellum has long been implicated in motor sequence learning and fine motor control (Ackermann, 2008; Bohland & Guenther, 2006; Doyon et al., 2002; Guenther, 2016), and prior work has documented greater recruitment of cerebellum for non-native compared to native sequences (Masapollo et al., 2021; Segawa et al., 2015). Few studies have examined the effects of cerebellar stimulation on *speech* motor control, but in a study on sensorimotor adaptation in speech, Lametti and colleagues (2018) found improved F1 compensation with cerebellum stimulation compared to sham; the authors concluded that tDCS to the cerebellum improves error correction (Lametti et al., 2018).

### Limitations

We acknowledge several limitations in the current study. An important limitation of this study is the absence of a pre-training baseline measure for the untrained clusters, which would offer a more direct measure of learning. Our primary measure of learning—performance differences between trained and untrained clusters—captures a specific aspect of training-related change but does not reflect improvement relative to a traditional pre-training baseline.

Furthermore, although the trained and untrained clusters were selected to be phonotactically similar in length and structure, they differed in segmental composition. This raises the possibility that observed differences in accuracy may partly reflect inherent production difficulty rather than training-specific improvement. Moreover, while untrained clusters were not intended to test generalization, their role as a control relies on the assumption that performance on these items remained stable. Given prior evidence that speech motor learning can generalize to phonologically distinct sequences (Cheng & Buchwald, 2021), we cannot rule out the possibility that untrained clusters also improved across the session. Together, these limitations constrain how confidently the trained–untrained difference score can be interpreted as a measure of learning. Future work should include pre- and post-training assessments of both trained and untrained clusters, and a clearly defined pre-stimulation baseline phase, to better isolate learning effects and account for potential generalization across stimuli.

Second, while the HD-tDCS system used here provides more focal stimulation than traditional tDCS systems, the close proximity of LpIFS to other critical cortical structures for speech, notably vPMC and portions of the inferior frontal gyrus ventral to pIFS, means that stimulation may have also affected these neighboring regions to a lesser degree (Figure 1). Therefore, the observed improvements in accuracy from pIFS stimulation may, at least in part, be due to the spread of tDCS effects to these neighboring areas. Further research is needed to distinguish the specific effects of tDCS on the phonological and motoric components of syllable production learning more clearly.

Another limitation of this study is the use of a single training session, unlike previous studies where training occurred over 2 days (Buchwald et al., 2019; Masapollo et al., 2021; Segawa et al., 2015, 2019). This longer training duration allows for the potential overnight consolidation of learning (cf. Krakauer & Shadmehr, 2006). Future work should explore whether the improvements in learning observed in the current study are retained over time.

Our study also lacked a double-blind design, which means the researchers were aware of condition assignment, which may have unintentionally introduced bias. We further acknowledge that the study was not pre-registered. While our hypotheses and analytic approach were theoretically motivated and determined prior to data collection, the absence of a formal pre-registration limits the transparency of our analytic decisions and increases the potential for bias in the interpretation of results. Future work would benefit from pre-registration to clearly delineate confirmatory from exploratory analyses and to enhance the reproducibility and rigor of findings.

Finally, in line with previous studies investigating non-native cluster learning (Masapollo et al., 2021; Segawa et al., 2015, 2019), we abstained from providing feedback during the experiment. While this decision facilitated standardization across participants, the absence of real-time feedback may have reduced the learning effects. Additionally, it may have amplified individual differences in perceptual abilities, with stronger perceivers more likely to detect deviations between their own speech and the target sample, while weaker perceivers may not. Since we did not directly measure perception, this remains a potential confounding factor (Ghosh et al., 2010).

## ACKNOWLEDGMENTS

We would like to thank our data collection team (Hannah Indiviglio, Mia Graziano, and Adanna Thomas) and the participants, without whom this research would not be possible.

## FUNDING INFORMATION

Frank H. Guenther, National Institute on Deafness and Other Communication Disorders (https://dx.doi.org/10.13039/100000055), Award ID: R01DC007683. Boston University, National Institute on Deafness and Other Communication Disorders (https://dx.doi.org/10.13039/100000055), Award ID: T32DC013017-07.

## AUTHOR CONTRIBUTIONS

**Hannah P. Rowe**: Conceptualization; Data curation; Formal analysis; Investigation; Methodology; Visualization; Writing – original draft. **Hilary E. Millar**: Conceptualization; Data curation; Formal analysis; Methodology; Validation; Writing – review and editing. **Rebecca M. Belisle**: Formal analysis; Investigation; Methodology; Writing – review and editing. **Tyler K. Perrachione**: Conceptualization; Funding acquisition; Methodology; Resources; Software; Supervision; Writing – review & editing. **Frank H. Guenther**: Conceptualization; Funding acquisition; Methodology; Project administration; Resources; Supervision; Writing – review and editing.

## COMPETING INTERESTS

Dr. Guenther receives royalties from MIT Press for his book *Neural Control of Speech*. There are no other conflicts of interest associated with this research.

## DATA AND CODE AVAILABILITY STATEMENT

The code supporting the findings of this study is available on GitHub at https://github.com/hrowe802. The data supporting the findings of this study are available on OSF at https://osf.io/jvdea/.

## Supplementary Material



## TECHNICAL TERMS

Transcranial direct current stimulation (tDCS): Noninvasive brain stimulation technique using weak electrical currents to modulate neural excitability and activity.
Sensorimotor adaptation: Process of adjusting motor actions based on sensory feedback to maintain accuracy in changing environmental conditions.
Epentheses: Insertion of extra sound within word, often to simplify pronunciation or fit phonological patterns.
