# Supplementary material for: Noninvasive Neurostimulation to the Inferior Frontal Sulcus or Cerebellum Improves Accuracy But Not Timing in a Speech Motor Learning Task
Source: Neurobiol Lang (Camb). 2025 Sep 24;6:NOL.a.18. doi: 10.1162/NOL.a.18 (PMC12534029; doi:10.1162/NOL.a.18)
Supplement: Supplementary file 1 [file nol-6-1-18-s001.pdf]

SUPPLEMENTARY MATERIALS

Table S1: Percentages of error types by onset versus offset (coda) consonant cluster and condition.

**Errors**	**LpIFS**		**RCbm**		**Sham**	
	Onset	***Offset***	***Onset***	***Offset***	***Onset***	***Offset***
Disfluency	0.23%	0.05%	0.26%	0.02%	0.46%	0.02%
Unrecognizable from target	0.72%	2.90%	0.70%	3.95%	1.25%	2.97%
Phoneme deletion/omission	10.30%	5.17%	9.41%	5.00%	11.48%	6.16%
Phoneme insertion	0.42%	3.45%	0.65%	5.01%	0.61%	4.41%
Consonant substitution	3.49%	7.21%	4.64%	8.16%	3.68%	7.36%
Incorrect phoneme order	0.23%	1.70%	0.31%	2.67%	0.33%	2.57%
Vocoid epenthesis	34.14%	0.32%	38.88%	0.46%	42.86%	0.41%
Incorrect voicing	5.71%	0.78%	7.58%	0.19%	5.86%	0.05%
Vowel substitution	0%	0.02%	0%	0.02%	0.03%	0.02%

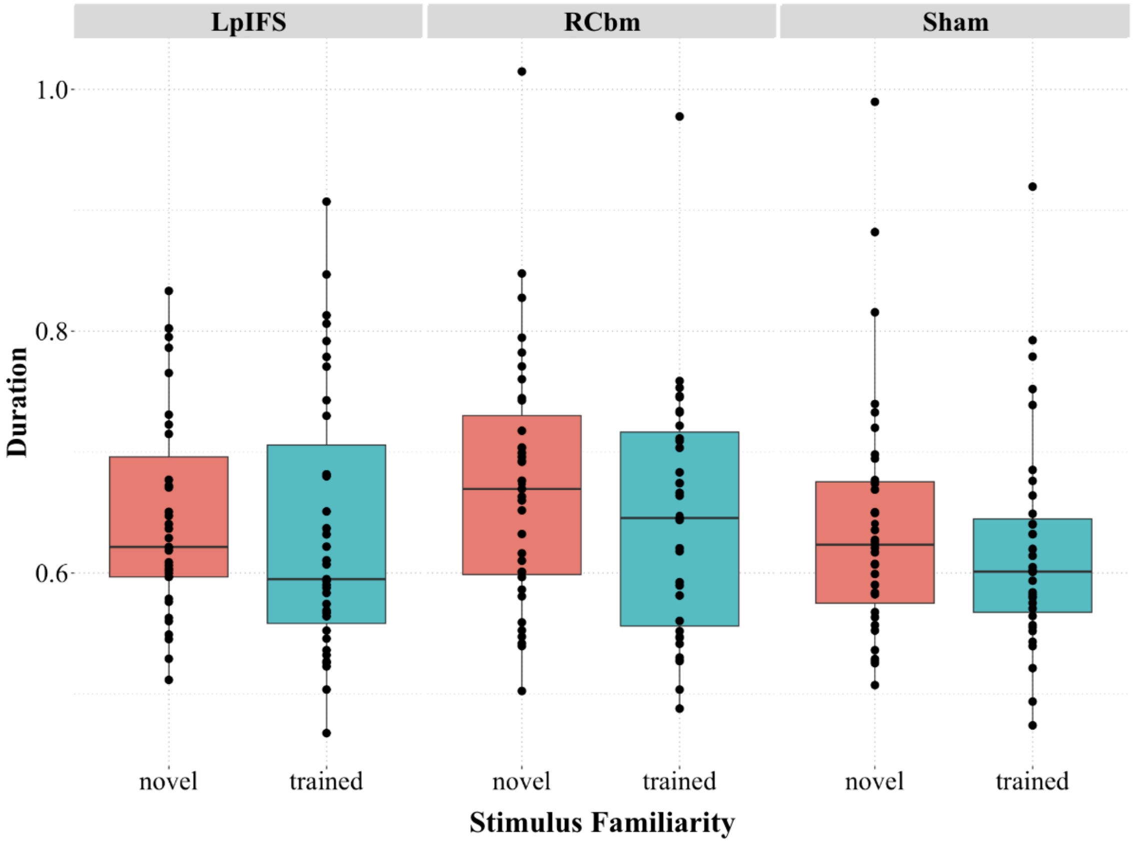

Figure S1. Boxplots showing the distributions of production durations (averaged across trials for each subject) for novel and trained stimuli in each stimulation condition.

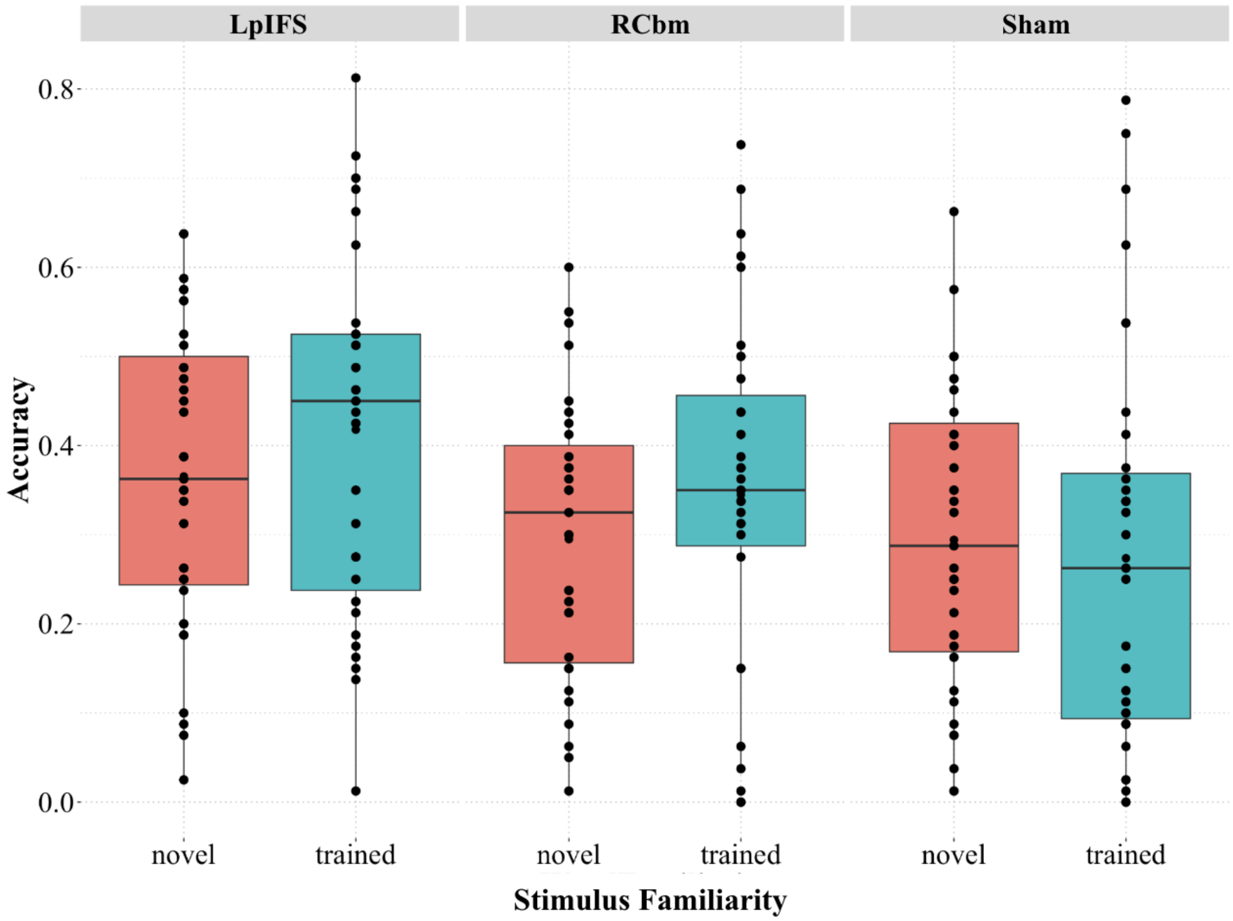

Figure S2. Boxplots showing the distributions of production accuracies (averaged across trials for each subject) for novel and trained stimuli in each stimulation condition.

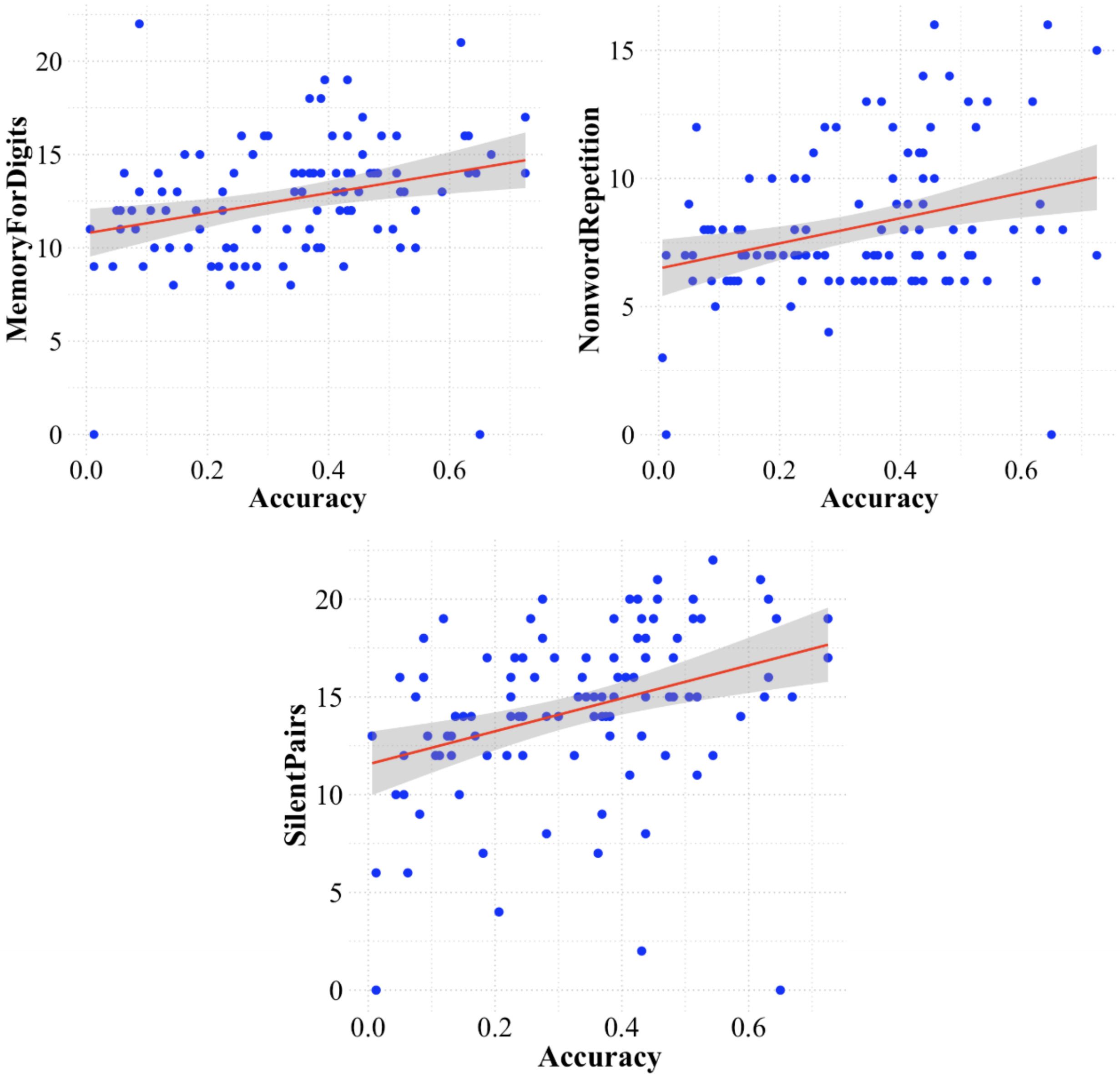

Figure S3. Scatterplots showing the significant correlations (Bonferroni-corrected alpha = 0.016) between working memory (WM) measures and outcome measures.
